# Alzheimer’s disease and multiple sclerosis: a possible connection through the viral demyelinating neurodegenerative trigger (vDENT)

**DOI:** 10.3389/fnagi.2023.1204852

**Published:** 2023-06-15

**Authors:** Marina S. Boukhvalova, Lorne Kastrukoff, Jorge C. G. Blanco

**Affiliations:** ^1^Sigmovir Biosystems, Inc., Rockville, MD, United States; ^2^Department of Medicine, The University of British Columbia, Vancouver, BC, Canada

**Keywords:** Alzheimer’s disease, multiple sclerosis, viral, HSV-1, demyelination, trigger, vDENT

## Abstract

Alzheimer’s disease (AD) and multiple sclerosis (MS) are two CNS disorders affecting millions of people, for which no cure is available. AD is usually diagnosed in individuals age 65 and older and manifests with accumulation of beta amyloid in the brain. MS, a demyelinating disorder, is most commonly diagnosed in its relapsing-remitting (RRMS) form in young adults (age 20–40). The lack of success in a number of recent clinical trials of immune- or amyloid-targeting therapeutics emphasizes our incomplete understanding of their etiology and pathogenesis. Evidence is accumulating that infectious agents such as viruses may contribute either directly or indirectly. With the emerging recognition that demyelination plays a role in risk and progression of AD, we propose that MS and AD are connected by sharing a common environmental factor (a viral infection such as HSV-1) and pathology (demyelination). In the viral DEmyelinating Neurodegenerative Trigger (vDENT) model of AD and MS, the initial demyelinating viral (e.g., HSV-1) infection provokes the first episode of demyelination that occurs early in life, with subsequent virus reactivations/demyelination and associated immune/inflammatory attacks resulting in RRMS. The accumulating damage and/or virus progression deeper into CNS leads to amyloid dysfunction, which, combined with the inherent age-related defects in remyelination, propensity for autoimmunity, and increased blood-brain barrier permeability, leads to the development of AD dementia later in life. Preventing or diminishing vDENT event(s) early in life, thus, may have a dual benefit of slowing down the progression of MS and reducing incidence of AD at an older age.

## Introduction

### MS pathogenesis, models, and treatment approaches

Multiple sclerosis (MS) is a demyelinating disorder of CNS affecting 2.3 million people worldwide (Wallin et al., 2019). It is most often diagnosed in individuals between 20 and 40 years of age (Howard et al., 2016). Historically, clinical subtypes of MS include clinically isolated syndrome, relapsing-remitting MS (RRMS), and primary and secondary progressive MS (Thompson et al., 2018). RRMS is the most common presentation of MS and is characterized by days to weeks of increased inflammation and demyelinated lesions in the white matter (Lassmann and Bradl, 2017). Depending on their location in the CNS, the lesions can lead to visual, sensory, motor, incoordination, neurocognitive, and bladder and bowel symptoms (Khan and Amatya, 2017). The acute clinical attack is followed by complete or partial recovery in patients, resulting from the resolution of inflammation and remyelination. Accumulating evidence suggests that relapsing vs. progressive MS phenotype is driven by “host factors,” most notably patient’s age, with younger patients displaying greater frequency of relapses and older patients more prone to having progressive phenotypes (Waubant et al., 2019).

The pathogenesis of MS includes attacks on myelinating glia [oligodendrocytes (OL)] in the CNS resulting in myelin degradation, axonal dysfunction, and neurodegeneration. The attack is thought to be immune-mediated, and is the basis for most disease modifying therapies (DMTs). Examples of approved treatments for MS include peptides found in myelin basic protein acting as a decoy for the attacking immune cells, a sphingosine-1-phosphate receptor modulator sequestering lymphocytes in lymph nodes, therapeutics preventing immune cell infiltration into the CNS, and β-interferon drugs (Derwenskus, 2011). While these treatments can slow progression of the disease, they are not capable of curing MS. Recently, remyelination-promoting therapies became a major focus of MS pharmacotherapy [reviewed in Melchor et al. (2019)].

There are 4 different animal models of demylination: (1) genetic/transgenic, (2) viral, (3) toxin-induced, and (4) autoimmunity-driven (Gudi et al., 2014; Boukhvalova et al., 2019; Torre-Fuentes et al., 2020). The latter two are most commonly used for the evaluation of MS therapeutics (Melchor et al., 2019). Toxin-induced demyelination is induced by feeding animals cuprizone, a copper chelator, or by injecting toxins like ethidium bromide or lysolecithin into the CNS. The autoimmunity-driven models (e.g., the model of Experimental Autoimmune Encephalomyelitis, or EAE) involve immunizing animals with myelin components to induce autoimmune attacks on myelin, or by passively transferring myelin-specific activated lymphocytes. These models have been very useful for understanding mechanisms of re-myelination and dissecting the role of various cell types in the process. However, neither toxin models nor EAE models reproduce MS as observed in humans, and may explain in part the failure of many immunomodulatory and neuroprotective treatment strategies in MS [reviewed in Rolfes et al. (2020) and Huntemann et al. (2021)].

### The role of viral infections in MS pathogenesis

The involvement of viral infections in triggering an acute attack in RRMS, potentially through a non-specific effect, has been suggested decades ago (Andersen et al., 1993; Panitch, 1994). A number of viruses including Epstein-Barr virus (EBV) and human herpes virus 6 (HHV-6) have been implicated in MS pathogenesis (Lindeberg et al., 1991; Haahr et al., 1992, 1995; Soldan et al., 1997; Munch et al., 1998; Virtanen and Jacobson, 2012; Bjornevik et al., 2022). However, how specific the role of these viruses is in acute attack of RRMS remains to be determined. A longitudinal study of 26 RRMS patients and 20 healthy controls that quantified EBV, HHV-6, cytomegalovirus (CMV) and herpes simplex virus 1 (HSV-1) DNA by PCR in PBMCs, showed that EBV and HHV-6 were detected in MS patients during acute attack and periods of remission, but also in healthy controls, with no significant differences between the MS patients and controls (Ferrante et al., 2000). In contrast, CMV and HSV-1 were detected only in MS patients, with HSV-1 DNA showing up only during an acute MS attack (Ferrante et al., 2000). This finding, together with the earlier suggestions (Lycke et al., 1996; Bergstrom, 1999; Ferrò et al., 2012), highlight HSV-1 as an important etiologic factor in triggering an acute attack in MS.

The role of HSV-1 in MS is difficult to model in laboratory animals. Prior to our recent work in cotton rats *S. hispidus*, multifocal demyelination, the main pathophysiologic feature of MS, could be induced by lip HSV-1 infection only in murine strains that carry inherent defects in complement system, macrophage function, and/or muscle repair (strains A/J, SJL/J, and PL/J) (Kastrukoff et al., 1987, 2012). These strains are used to study developmental defects, epilepsy, spontaneous tumorigenesis, myopathy, and/or autoimmunity, all of which may affect CNS manifestations. Cotton rats *S. hispidus* are not prone to these disorders and, instead, have proven to be a reliable translational model of human viral diseases (Boukhvalova et al., 2009, 2015, 2018, 2022). The lip HSV-1 infection in *S. hispidus* delivered by abrasion caused multifocal demyelination in the CNS, followed by remyelination and formation of MS-like plaques (Boukhvalova et al., 2019). Virus antigens were detected in association with demyelinated lesions, suggesting a direct effect of viral infection/presence in the brain. Involvement of thalamus was noted, with perivascular cuffing and potential demyelination developing in the area. In human MS cases, involvement of the thalamus has been associated with a variety of clinical manifestations, including fatigue, movement disorders, pain, and cognitive impairment (CI) (Amin and Ontaneda, 2020). A recent study of brain samples from chronic progressive MS cases showed that active MS lesions were populated by CD8 + tissue-resident memory T cells with signs of reactivation and infiltration into the brain parenchyma (Fransen et al., 2020), possibly as a recall response to viral infection/reactivation in the CNS. Accumulating evidence, therefore, points to an important role of viral infections/reactivations in MS pathogenesis and etiology.

### AD pathogenesis and current treatment approaches

Alzheimer’s disease (AD) is a disorder that affects cognitive function and memory that can lead to dementia. Dementia caused by AD is diagnosed usually in people age 65 and older, and affects an estimated 6.7 million Americans (Alzheimer’s disease facts and figures, 2023). The main pathologic findings in AD are the extracellular amyloid plaques and the intracellular Tau neurofibrillary tangles (Yiannopoulou and Papageorgiou, 2020). AD pathophysiology is based on the “amyloid hypothesis,” where cleavage of the large amyloid precursor protein (APP) into protease-resistant peptide fibrils results in formation of beta amyloid (Aβ) plaques. The process triggers neurotoxicity, local inflammation, oxidation, excessive glutamate (excitotoxicity), and Tau hyperphosphorylation. Tau is a microtubule-associated protein that helps neuronal transport system and stabilizes growing axons. Abnormally hyperphosphorylated Tau forms intra-neuronal tangles composed of insoluble fibrils (Anand et al., 2017). Accumulating neuronal damage leads to deficiencies and imbalance between different neurotransmitters (e.g., acetylcholine, dopamine, serotonin) and associated cognitive deficiencies (Yiannopoulou and Papageorgiou, 2020). Treatments approved for AD have historically been purely supportive and aimed at counterbalancing the neurotransmitter imbalance. They include acetylocholinesterase inhibitors and an NMDA-receptor open-channel blocker that affects glutamatergic transmission (Yiannopoulou and Papageorgiou, 2013; Cummings et al., 2019). Multiple clinical trials of disease modifying treatments (DMT) with drugs that target amyloid-related mechanisms or associated inflammation have met with mixed results (Yiannopoulou and Papageorgiou, 2020). In the past 2 years, the FDA has approved two drugs for AD treatment: aducanumab and Leqembi (lecanemab-irmb). Both are monoclonal antibodies targeting Aβ, shown to reduce appearance of amyloid plaques, and both have advanced through the FDA accelerated approval system. However, there are concerns over efficacy and serious adverse events. One study of aducanumab identified cerebral edema or hemorrhage in 41% of patients in the study (Salloway et al., 2022). The process of accelerated approval does indicate a dire need for effective AD therapeutics at the time when the elderly population is increasing worldwide (Owolabi et al., 2023). The lack of success of a number of amyloid- and immune-targeting AD therapeutics in recent years (reviewed Mullane and Williams, 2020) argues for a better understanding of AD etiology and pathogenesis.

### New developments in the AD field: the role of viral infections, myelin damage, and immune response

The number of publications supporting a role for HSV-1 in pathogenesis of AD has steadily increased and has recently been reviewed (Itzhaki, 2017, 2021). In brief, HSV-1 can enter the CNS and reside there in latent form. Individuals with the type 4 allele of the apolipoprotein E gene (APOE-ε4) are at increased risk of AD development after HSV-1 infection (Wu et al., 2020). In a Taiwanese study of 8,362 subjects aged ≥ 50 years, newly diagnosed with HSV (HSV-1 or HSV-2), and exhibiting severe symptoms of herpes labialis and/or genitalis, an increased risk of 2.56-fold of developing dementia in a 10-year follow up compared to controls was identified. The risk was reduced in patients who received antiherpetic medications (Tzeng et al., 2018). Further support comes from *in vitro* studies where HSV-1 was reported to induce accumulation of Aβ in cultured neurons (De Chiara et al., 2010; Piacentini et al., 2011) and to promote Tau hyper-phosphorylation (Zambrano et al., 2008; Wozniak et al., 2009). A recent study in mice infected with HSV-1 by lip abrasion showed that repeat reactivation of virus following thermal stress led to progressive accumulation of AD biomarkers, including Aβ and abnormal Tau, and development of cognitive deficits (De Chiara et al., 2019). Apart from HSV-1, other viruses, including varicella zoster virus (VZV), EBV, CMV, and HHV-6, have been linked to dementia, but for at least some of them it is not clear whether neurodegeneration develops as a result of direct virus involvement or an indirect effect on inflammation that reactivates HSV-1 (Cairns et al., 2022).

Although AD has long been considered a disease of gray matter, recent neuroimaging studies have identified micro- and macro-structural changes in the white matter that could contribute to risk and progression of AD, resulting in a shift of focus in AD research toward myelin and oligodendrocytes [reviewed in Nasrabady et al. (2018)]. It has also been shown that several AD-relevant pathways overlap significantly with remyelination pathways that contribute to myelin repair by encouraging oligodendrocyte proliferation. Importantly, amyloid, Tau, and ApoE, previously defined as therapeutic targets of AD, contribute to both remyelination and AD progression (Papuć and Rejdak, 2018). Aggregated Aβ 42 and neurofibrillary tangles may not only be responsible for neuronal loss but can also induce myelin damage and oligodendrocyte death (Papuć and Rejdak, 2018). The impairment in the formation of myelin sheath can even precede Aβ and Tau pathologies in AD (Couttas et al., 2016; Papuć and Rejdak, 2018). The contribution of immune-mediated mechanisms to pathogenesis of AD is also gaining increased recognition. Dysregulation of monocyte subsets, accumulation of neutrophils in the CNS, depleted and/or dysfunctional regulatory T cells (Tregs), and brain damage mediated by CD8 + T cells have now been documented in both AD and MS cases [reviewed in Rossi et al. (2021)].

### The vDENT model

**FIGURE 1:**
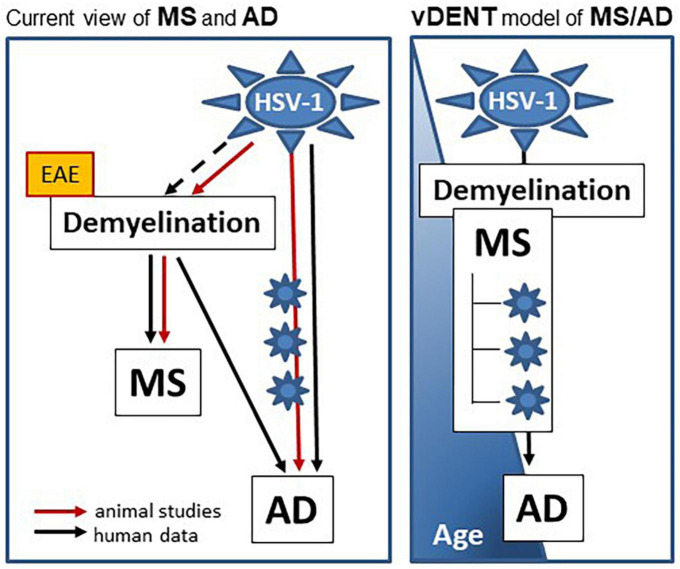
vDENT (viral demyelinating neurodegenerative trigger) model of AD and MS. vDENT model of MS/AD (on the **right**) is based on the current view of MS and AD (on the **left**). In this current view, HSV-1 contributes to AD in humans and animal models through repeated reactivation of virus in the nervous system (blue star symbols on the red line). Contribution of HSV-1 to MS in humans is not entirely clear (dashed black line), however, it’s been demonstrated in animals (cotton rats, solid red line). Although demyelination is central to the pathogenesis of MS in both humans and animals, many therapies tested in the non-infectious EAE models (yellow box) have failed to show efficacy in humans. Not only is demyelination central to MS, it is also recognized as being important for risk and progression of AD in humans. A model is proposed on the right in which MS and AD are linked to the same vDENT event early in life, which can lead to development of AD later on.

The scientific fields of MS and AD appear to be rapidly changing, in part because of a lack of success of a number of immune- or amyloid-targeting therapeutics developed on the basis of an earlier understanding of the pathogenesis of these diseases (Mullane and Williams, 2020; Rolfes et al., 2020; Huntemann et al., 2021). It is becoming clear that MS and AD, albeit disparate in regard to the timing of their diagnosis and the extent of cognitive impairment, share a number of important similarities, such as the contribution of herpesvirus infections, demyelination, and immune dysregulation. The potential role of an infectious etiology in MS and AD is becoming more focused. Members of the family Herpesviridae including HSV-1, EBV, CMV, HHV-6, VZV (and others) have long been suspected of playing a role, but their involvement has never been proven. Recently, a contribution of HSV-1 to AD has been acknowledged, while a similar interest in the contribution of herpesviruses to MS is increasing. We would like to propose that MS and AD are connected, share a viral infection as an environmental trigger, and demyelination as a common factor in pathogenesis. We propose the viral DEmyelinating Neurodegenerative Trigger (vDENT) model of AD and MS (Figure 1) where the initial viral infection (e.g., HSV-1) and ensuing demyelination provoke the first episode of MS-like disease early in life, with subsequent viral reactivations and associated immune/inflammatory attacks leading to appearance of RRMS-like disease, with periods of symptomatic disease coinciding with virus reactivation/demyelination episodes and remission brought on by remyelination and resolution of immune/inflammatory reaction. The CNS damage accumulating during the repeated reactivation episodes would lead to amyloid dysfunction, which, combined with the potential virus progression deeper into the CNS, inherent remyelination defects developing in older age (Barbosa et al., 2019; Dimovasili et al., 2023), and altered immune and blood-brain barrier function (Mooradian, 1988; Ransohoff, 2023), would bring on AD-like cognitive defects. It is also possible that neurodegenerative damage accumulates in the absence of symptomatic reactivation episodes (MS forms other than RRMS), that demyelination becomes less pronounced with subsequent reactivation events, and/or that immune dysfunction plays a bigger role during the later stages of MS that occur at an older age, manifesting the prevalence of progressive MS form over RRMS in the elderly (Waubant et al., 2019; Ransohoff, 2023).

The vDENT model of MS/AD stipulates that developing MS after viral infection early in life can lead to symptomatic AD in old age, and that preventing/lessening MS can reduce incidence of AD. More intricately, it suggests that the pre-symptomatic phase of AD, which may span decades and appear well before the cognitive defects develop (Braak et al., 2011; Braak and Del Tredici, 2014, 2015), may overlap with the mid- or late- stages of MS and represent a progression of the same pathophysiologic mechanism initiated by viral infection. The recent demonstrations that HSV-1 can directly cause Tau pathology [reviewed in Harris and Harris (2018) and Duarte et al. (2019)], and that Tau defects appear during the first decades in life, while amyloid abnormalities occur at an older age (Braak and Del Tredici, 2015), support the progressive nature of viral-induced CNS neurodegeneration. The connection of both AD and MS to demyelination, the critical role demyelination can play in initiation (and potentially relapsing nature) of MS, and overlap of demyelination and AD-critical pathways, further support the link between AD, MS, and viral infections that can cause demyelination. Importantly, during the earlier stages of AD, Tau defects are found not in the cortex but in the neurons of the brainstem (BST) (Braak and Del Tredici, 2015), the same place where the first demyelinating lesions appear after the lip HSV-1 infection. In both HSV-1 infected cotton rats and in susceptible murine strains, demyelinated lesions after the initial HSV-1 infection progress in the sequence BST > cerebellum > cerebral hemispheres (Kastrukoff et al., 1992, 2012; Boukhvalova et al., 2019).

Multiple sclerosis is very heterogeneous in its clinical course, clinical severity and outcome, pathological appearance, MRI appearance, and response to therapy. It is possible that vDENT model applies only to a subset of MS cases. It is also likely that the model applies to a small fraction of all herpesvirus infections, as seroprevalence of some of them (e.g., HSV-1) can be as a high as 90% in developed countries (Roizman and Knipe, 2001; Whitley and Roizman, 2001). The selection may depend on the ability of herpesviruses to induce CNS demyelination under certain conditions. One example here may include a specific age at which the first (acute) infection occurs, and whether it happens in a susceptible child/adolescent or an adult. Our studies in cotton rats indicate that demyelination in the CNS after lip HSV-1 infection occurs prevalently in young animals, when brain plasticity is still high, and that demyelination and disease in animals infected with HSV-1 for the first time as “adults” are less pronounced (Boukhvalova et al., 2019, 2022). This finding is important as it may indicate that vDENT hypothesis of MS/AD connection applies specifically to select pediatric-onset MS cases (Thompson et al., 2018). It is also possible that the model applies to a subset of MS patients with detectable lesions in trigeminal root entry zone (TREZ) [about 10% (Sugiyama et al., 2015)], as TREZ is a portal often utilized by herpesvirus infections. Overall, only a fraction of herpesvirus-infected individuals may go on to develop CNS demyelination, MS, and subsequently AD.

The direct progression from MS to AD has not been proposed before, possibly because of the reduced life expectancy in MS patients in the past compared to the general population (Lunde et al., 2017; Leadbetter et al., 2023), because of so many diverse forms/manifestations of MS, because remyelinated lesions are often difficult to image (potentially precluding detection of both MS and AD pathology in the same autopsy samples), and/or because of the lack of systematic studies searching for the causative association between MS and AD. It is known, however, that cognitive dysfunction develops in about half of MS patients (Sumowski et al., 2018), potentially influenced by genetics and lifestyle. As the survival gap between MS patients and general population appears to be receding due to progress in disease management (Leadbetter et al., 2023), detection of MS to AD progression could become easier in future studies designed to detect markers of both diseases in respective patient cohorts of all ages, taking into account the evolving nature of these diseases. The overlap may be easier to correlate to viral markers during the late MS - early (preclinical) AD in patients who are younger, as the disease may progress to the more immune-mediated mechanisms and the frequency of MS relapses (and coincidentally detectable viral markers) may reduce with advancing age (Waubant et al., 2019).

It is possible that in those individuals who are genetically susceptible to developing MS (with or without influence of additional environmental factors), the initial demyelinating event and later reactivations of virus can trigger a complex abnormal immune reaction directed at myelin and myelinating cells (Miller et al., 2001; Vanderlugt and Miller, 2002). With repeated viral reactivation and damage to the CNS, breaks in tolerance, epitope spread, bystander activation, and molecular mimicry will evolve and begin to take over from viral reactivation as the driving force behind the disease (Miller et al., 1997, 2001). Eventually MS can be established as an autoimmune disease. In those individuals who are genetically susceptible to developing AD, the initial demyelinating event and later reactivations of virus can trigger an abnormal immune reaction directed at neuronal cells (Jamieson et al., 1991; Itzhaki et al., 1997; Mori, 2010; Rossi et al., 2021). It can be a secondary event with the primary event being virus taking over neuronal function and giving rise to the toxins that eventually result in abnormal Tau proteins and amyloid bodies (Duarte et al., 2019). The proposed connection between MS and AD through the common viral demyelinating trigger, therefore, may be complicated, but is nevertheless important as it suggests that therapeutics capable of slowing down progression of MS may also be able to reduce incidence of AD at an older age.

## Conclusion

Recently, a theory that Tau pathology is an initiating event leading to sporadic Alzheimer’s disease has been proposed (Arnsten et al., 2021). This theory is partly based on the fact that Tau abnormalities are first detected in childhood, while amyloid abnormalities do not show up until an older age (Braak and Del Tredici, 2015). vDENT theory, and the fact that HSV-1 infection itself can cause Tau abnormalities, fits this “Tau-first” hypothesis very well and takes it one step further by suggesting that the first Tau abnormalities in children and/or young adolescents are caused by the first encounter with HSV-1 (or other demyelinating viruses) at an age when the brain is more susceptible to virus-induced demyelination and when the immune system is still naïve to these viruses. vDENT theory of MS/AD connection suggests that, in some cases, as the child/adolescent becomes an adult, and then an elderly, inherent aging-related deficiencies may contribute to the transition from MS to AD, including defects in remyelination mechanisms (Barbosa et al., 2019; Dimovasili et al., 2023), increased permeability of blood-brain barrier (Mooradian, 1988), and propensity for autoimmunity (Ransohoff, 2023). Historical arguments of immune and inflammatory mechanisms contributing to AD and MS pathogenesis, therefore, are not excluded by the vDENT theory. On the contrary, they are a crucial part of it that should be incorporated through the lenses of antigen-specific local mechanisms in brain parenchyma that may not have been considered before.

## Data availability statement

The original contributions presented in this study are included in the article/supplementary material, further inquiries can be directed to the corresponding author.

## Author contributions

MB conceptualized the model and performed the literature review and information interpretation. LK provided a critical revision. MB, LK, and JB were involved in manuscript preparation. All authors contributed to the article and approved the submitted version.
